# Ca^2+^ Dyshomeostasis Disrupts Neuronal and Synaptic Function in Alzheimer’s Disease

**DOI:** 10.3390/cells9122655

**Published:** 2020-12-10

**Authors:** John McDaid, Sarah Mustaly-Kalimi, Grace E. Stutzmann

**Affiliations:** 1 Center for Neurodegenerative Disease and Therapeutics, Rosalind Franklin University of Medicine and Science, 3333 Green Bay Rd., North Chicago, IL 60064, USA; john.mcdaid@rosalindfranklin.edu (J.M.); sarah.mustaly@my.rfums.org (S.M.-K.); 2 School of Graduate and Postdoctoral Studies, Rosalind Franklin University of Medicine and Science, 3333 Green Bay Rd., North Chicago, IL 60064, USA; 3 Chicago Medical School, Rosalind Franklin University of Medicine and Science, 3333 Green Bay Rd., North Chicago, IL 60064, USA

**Keywords:** calcium, synaptic, glutamate, nicotinic receptors, mitochondria, autophagy, lysosome

## Abstract

Ca^2+^ homeostasis is essential for multiple neuronal functions and thus, Ca^2+^ dyshomeostasis can lead to widespread impairment of cellular and synaptic signaling, subsequently contributing to dementia and Alzheimer’s disease (AD). While numerous studies implicate Ca^2+^ mishandling in AD, the cellular basis for loss of cognitive function remains under investigation. The process of synaptic degradation and degeneration in AD is slow, and constitutes a series of maladaptive processes each contributing to a further destabilization of the Ca^2+^ homeostatic machinery. Ca^2+^ homeostasis involves precise maintenance of cytosolic Ca^2+^ levels, despite extracellular influx via multiple synaptic Ca^2+^ channels, and intracellular release via organelles such as the endoplasmic reticulum (ER) via ryanodine receptor (RyRs) and IP_3_R, lysosomes via transient receptor potential mucolipin channel (TRPML) and two pore channel (TPC), and mitochondria via the permeability transition pore (PTP). Furthermore, functioning of these organelles relies upon regulated inter-organelle Ca^2+^ handling, with aberrant signaling resulting in synaptic dysfunction, protein mishandling, oxidative stress and defective bioenergetics, among other consequences consistent with AD. With few effective treatments currently available to mitigate AD, the past few years have seen a significant increase in the study of synaptic and cellular mechanisms as drivers of AD, including Ca^2+^ dyshomeostasis. Here, we detail some key findings and discuss implications for future AD treatments.

## 1. Ca^2+^ Dysregulation and Synaptic Defects in AD

The synapse, as the primary site of communication between neurons, plays a vital role in the transmission of neuronal impulses and information, and for encoding of learning and memory, all of which are affected in Alzheimer’s disease (AD). AD, as a progressive neurodegenerative disease, is characterized by Ca^2+^ dysregulation i.e., a “calciumopathy” [1] and synapse loss, i.e., a “synaptopathy” [2], with emerging evidence for a causal link between the two. Synaptic density is decreased in post-mortem brain tissue from AD patients [3,4], and while amyloid plaques have been implicated in AD related synaptic loss, synaptic deficits occur prior to and in the absence of amyloid plaques [5], and may also be due to Ca^2+^ dysregulation, an effect which is exhibited in presymptomatic AD mouse models [6,7,8,9]. Ca^2+^ dysregulation is characterized by exaggerated Ca^2+^ responses to synaptic and other stimuli, as well as abnormal Ca^2+^ homeostasis [10,11], both of which may result in elevated resting cytosolic Ca^2+^, an effect which is observed in AD and older non-AD rodent models [12,13,14,15,16].

### 1.1. Ca^2+^ Dysregulation Disrupts Synaptic Networks in AD

Synapses are unique Ca^2+^ entry points in the neuronal architecture, expressing both pre- and postsynaptic Ca^2+^ channels/receptors, including presynaptic RyRs, N/P/Q voltage gated Ca^2+^ channels (VGCCs), α7 nicotinic acetylcholine receptors (α7 nAChRs), and postsynaptic L-type VGCCs, RyRs and NMDA receptors (NMDARs). NMDARs in particular are one of the most well characterized postsynaptic glutamate receptors, with a high Ca^2+^ permeability and an established role in hippocampal synaptic plasticity [17]. At hyperpolarized potentials, NMDARs are blocked by Mg^2+^, but postsynaptic depolarization results in removal of Mg^2+^ block, and receptor disinhibition. Repeated NMDAR activation enhances postsynaptic Ca^2+^ entry, an effect which is facilitated by RyRs through Ca^2+^-induced-Ca^2+^ release (CICR), thus driving increased postsynaptic AMPA receptor (AMPAR) expression and subsequent synaptic long-term potentiation (LTP). This dual role of the NMDAR as coincidence detector and postsynaptic Ca^2+^ entry channel makes it uniquely positioned to mediate synaptic potentiation resulting from concurrent pre- and postsynaptic activation, thus forming a mechanistic basis for Hebbian plasticity and associative learning.

Paradoxically, NMDARS, as well as playing a role in synaptic plasticity, may also play a role in synaptic loss [18] and cell death [19]. The role of NMDARs in the deleterious effects of AD is further illustrated by the efficacy of the NMDAR antagonist memantine as a treatment for moderate to severe AD [20,21]. Interestingly, the clinical efficacy, or lack thereof, of specific Ca^2+^ channel antagonists could serve as a useful pointer for a role for those Ca^2+^ channels in the pathophysiology of AD, with the failure of large scale clinical trials for L-type VGCC antagonists in particular, contrasting with the positive effects of memantine [22]. AD is characterized by synaptic loss [2,4,23], including loss of synaptic terminals and dendritic spines [4], and similar dendritic spine loss is accompanied by impaired synaptic transmission and plasticity in animal models of AD [8,24,25,26,27,28,29]. Although the cause of synaptic loss in AD is not fully understood, it is thought to be associated with increased ER- Ca^2+^ release within spines [8,9,30,31], and at later disease states, toxic soluble Aβ species [32], resulting in hippocampal dendritic spine loss via NMDAR activation [33,34,35]. In contrast to Aβ, synaptic effects of abnormal tau expression have not been as extensively studied, however, a few recent studies have implicated effects of tau on VGCC function and synaptic signaling [36,37,38]. Specifically, tau accumulation may lead to synaptic loss and impairment of synaptic function via activation of calcineurin [39].

Hippocampal and cortical neurites in close proximity to amyloid plaques demonstrate Ca^2+^ hyperactivity in vivo, in presymptomatic AD mice, an effect which is blocked by AMPA receptor and NMDAR antagonists, suggesting that this hyperactivity is synaptically driven [6,7]. Although Ca^2+^ hyperactivity occurred mainly in the vicinity of insoluble dense core plaques, these plaques are also surrounded by soluble Aβ [40,41], which causes similar hyperactivity in WT mice [6]. Furthermore, plaque proximity has been reported to have no effect on evoked dendritic RyR and VGCC mediated Ca^2+^ signaling in AD mice [42], raising the possibility that some of the hyperactivity observed may be due to a presynaptic mechanism. Indeed, the presynaptic Ca^2+^ hyperactivity observed in an AD mouse model was inhibited by the sarcoplasmic endoplasmic Ca^2+^-ATPase (SERCA) pump inhibitor cyclopiazonic acid, indicating that this Ca^2+^ hyperactivity is driven by activation of presynaptic Ca^2+^ stores [43]. In contrast to reports of synaptically driven hyperexcitability in vivo, studies carried out using acute brain slices from AD mice demonstrate decreased basal hippocampal synaptic transmission, sometimes accompanied by increased paired-pulse ratio of evoked field potentials, indicative of decreased presynaptic glutamate release probability [29,44,45]. In addition, the membrane afterhyperpolarization mediated by activation of postsynaptic Ca^2+^ activated SK2 channels is increased in a 3xTg AD mouse model [46], leading to decreased postsynaptic membrane excitability and possible decreased synaptic transmission. It should also be noted that the decreased hippocampal synaptic transmission recently observed in a 5xTg AD mouse model was coupled with increased postsynaptic membrane excitability, due to an RyR2 mediated decrease in A-type K^+^ current (I_A_) [47], thus further illustrating the complexity of synaptic effects observed in AD mouse models. It is also noteworthy that the in vivo hyperexcitability studies mentioned above were conducted in animals anesthetized using the volatile inhalational anesthetic isoflurane. As isoflurane has been shown to result in increased cytosolic Ca^2+^ in hippocampal neurons [48], possibly due to IP_3_ receptor activation [49], effects which are exaggerated in AD mice [50], isoflurane anesthesia could be a potential mediator of the Ca^2+^ hyperexcitability observed in vivo in AD mice.

While the last two decades have seen a large increase in the number of studies using AD mouse models, a more recent development has been in the use of human induced neurons (HiNs), which are neurons derived from tissue samples taken from patients, to study synaptic transmission [51,52,53]. In a recent study, an increased frequency of spontaneous excitatory postsynaptic current (EPSC’s) was observed in AD derived HiNs, indicating an impulse-independent spontaneous increase in presynaptic glutamate release probability which is consistent with findings in human and animal studies [52]. Studies in patients with mild cognitive impairment have demonstrated hippocampal hyperactivity and decreased hippocampal volume [54,55], indicating a possible correlation between increased hippocampal activity and neurodegeneration.

More recently, proteomics has emerged as a method that allows for high throughput analysis of protein expression in small tissue samples [56], including post-mortem brain tissue from AD patients [57,58], and which has allowed for the study of changes in the interaction between presynaptic proteins [59], including SNAP25 and syntaxin [60]. Increased SNAP25 and syntaxin interaction results in reduced glutamatergic synaptic transmission [61,62] and decreased interaction between these proteins has been observed in the brains of AD patients, along with decreased levels of Complexin II [63], effects which would be expected to result in increased excitatory synaptic transmission [64,65]. SNAP25 has also been shown to negatively interact with presynaptic VGCCs to control presynaptic Ca^2+^ and affect neurotransmitter release [66,67], and the demonstrated therapeutic efficacy of putative AD medications such as levetiracetam, which targets presynaptic VGCCs [68,69], suggests that presynaptic Ca^2+^ channels could serve as a therapeutic target for AD.

### 1.2. Acetylcholine Signaling and α7nAChR Function in AD

nAChRs are essential for normal cognitive function [70,71], and this family of receptors includes the highly Ca^2+^ permeable homomeric α7nAChR isoform [72,73]. α7nAChRs are expressed throughout the septo-hippocampal circuit, both on medial septal nucleus/diagonal band cholinergic neurons [74], and also in the hippocampus. Cholinergic neurons in particular show significant degeneration in the course of AD [75,76], and this has resulted in development of medications to enhance cholinergic transmission, presumably through the activation of postsynaptic nAChRs. Hippocampal α7nAChRs are expressed presynaptically on mossy fiber terminals [77], and postsynaptically on CA1 interneurons [78,79], with activation of both resulting in Ca^2+^ influx [79,80,81,82,83]. Nicotine enhances hippocampal excitatory synaptic transmission via activation of α7nAChRs on mossy fiber terminals in the hippocampal CA3 region [80,81,84] and activation of CA1 α7nAChRs facilitates hippocampal LTP [85].

Aβ binds with high affinity to α7nAChRs [86,87], resulting in noncompetitive block of α7nAChR function, including at presynaptic α7nAChRs [88]. Cortical and hippocampal α7nAChR expression is reduced in AD patients [89,90] and AD mice [91] and Aβ binding to α7nAChRs results in the endocytosis of the Aβ α7nAChR complex with resulting accumulation within the lysosomal compartment [92]. Aβ binding to α7nAChRs results in Ca^2+^ influx, both in oocytes and presynaptic terminals in hippocampus [93,94]. Low micromolar concentrations of Aβ trigger glutamate release in the hippocampal dentate gyrus, CA3 and CA1 subfields via α7nAChRs [95] and picomolar concentrations of Aβ enhance hippocampal LTP via α7nAChRs [96]. In addition, α7nAChR activation rescues LTP deficits in hippocampal slices taken from Aβ infused rat brains, and Aβ treated hippocampal slices [97,98] and chronic treatment with an α7nAChR agonist restores cognition in AD mice [99]. Cells treated with the acetylcholinesterase inhibitor donepezil, used clinically in the treatment of AD, also showed reduced glutamate NMDAR mediated Ca^2+^ influx, an effect which was blocked by an α7nAChR antagonist [100]. Galantamine, also an acetylcholinesterase inhibitor, has been shown to positively modulate human α7nAChRs expressed in xenopus oocytes, thus allowing for a dual effect of increased synaptic acetylcholine and α7nAChR potentiation [101].

Although a role for α7nAChRs in the etiology of AD has not been established, many animal studies have demonstrated cognitive enhancing effects of compounds targeting α7nAChRs [102], including the α7nAChR partial agonist EVP-6124, which has also been shown to enhance cognition in patients with mild to moderate AD [103]. EVP-6124 and the α7nAChR positive allosteric modulator AVL-3288 have been shown to be well tolerated in patients [104,105], but some concerns exist about effects of potentiation of α7nAChR mediated Ca^2+^ effects in AD. In addition to α7nAChR mediated Ca^2+^ influx, activation of α7nAChRs triggers CICR via ryanodine sensitive Ca^2+^ stores [106], including at presynaptic α7nAChRs on hippocampal mossy fiber terminals [107], which are known to have strong RyR expression [108]. As RyR mediated CICR may be increased in AD, positive allosteric modulation of α7nAChRs may facilitate pre- and postsynaptic Ca^2+^ overload via already increased RyR function [109], possibly exacerbating AD related synaptic deficits. Based on these studies, the use of α7nAChR compounds in the treatment of cognitive impairment and AD looks promising, but caution should be exercised regarding the use of drugs which result in overt α7nAChR potentiation.

### 1.3. Potential Therapies for the Treatment of Synaptic Ca^2+^ Dysregulation in AD

As of now, there are only two FDA-approved classes of drugs used in the symptomatic treatment of AD: the noncompetitive NMDA antagonist memantine, and the acetylcholinesterase inhibitors, donepezil, galantamine and rivastigmine, with both classes of drugs having a synaptic site of action. Although both memantine and donepezil have been shown to be moderately effective in the treatment of AD symptoms, there is an urgent need for disease-modifying approaches, which currently requires the identification of novel compounds and receptor targets at the pre- or postsynaptic level. While a number of studies have identified promising small molecules targeting NMDARs [19,51], α7nAChRs [85,99], RyRs [110] and SERCA [111], few have made it past the preclinical stage of testing. In addition, the smoking cessation medication varenicline, which is an agonist at the α7nAChR [112], has been tested as a treatment for AD, but without any observed beneficial effects in patients [113]. Despite its failure, the clinical trial for varenicline illustrates the use of existing FDA approved medications as a strategy in the treatment of AD, bypassing many of the arduous and expensive aspects of drug development. The RyR modulator dantrolene (Ryanodex) is an FDA approved medication that has been shown to be effective in reversing many of the synaptic and cognitive effects seen in mouse models of AD [8,114,115,116], and has good CNS penetration when given orally or by nasal administration [117]. In addition, the clinically used L-type VGCC inhibitor isradipine has been shown to be neuroprotective in an AD mouse model [22], as has the beta-blocker carvedilol [47], however results from a recent large clinical study suggested no benefit of the VGCC antagonist nilvadipine as a treatment for AD [118]. Although the failure of large scale clinical trials for VGCC inhibitors as a treatment for AD has resulted in diminished enthusiasm for their use, the antiepileptic drug levetiracetam, which inhibits presynaptic VGCCs [68], has been shown to be beneficial in AD patients [69] and clinical trials for its use in the treatment of AD are ongoing [119]. The relative success of levetiracetam, along with the well documented failure of clinical trials targeting amyloid, strengthens the case for the use of synaptically targeted drugs in the treatment of AD and argues for the testing of FDA-approved medications as an important therapeutic strategy in the treatment of AD.

### 1.4. ER Ca^2+^ Channels in Synaptic Dysregulation in AD

Cytosolic Ca^2+^ levels are tightly regulated and maintained at low nM concentrations, despite a much higher extracellular Ca^2+^ concentration, and similarly elevated Ca^2+^ levels within intracellular organelles such as the ER. The ER is located throughout the cell, including pre- and postsynaptically, at synaptic terminals and dendritic spines respectively. RyRs, as well as having a role in gating ER Ca^2+^, are sensitive to changes in cytosolic Ca^2+^ through the process of CICR, with increases in postsynaptic Ca^2+^ resulting in RyR activation and release of ER Ca^2+^ into the cytosol. CICR has the effect of amplifying postsynaptic Ca^2+^ generated from influx via Ca^2+^ permeable receptors/ion channels such as NMDA receptors (NMDAR) and voltage gated Ca^2+^ channels (VGCCs), and RyR mediated CICR is upregulated in neurons in 3xTg AD mice [9]. Although RyR mediated amplification of postsynaptic Ca^2+^ allows for a large rapid increase in cytosolic Ca^2+^, this increased Ca^2+^ is usually rapidly removed from the cytosol, against a concentration gradient, by Ca^2+^ ATPases including the SERCA pump, which is also located on the ER membrane. The ER membrane also expresses IP_3_ receptors, although these are not thought to be synaptically expressed [120].

**Figure 1:**
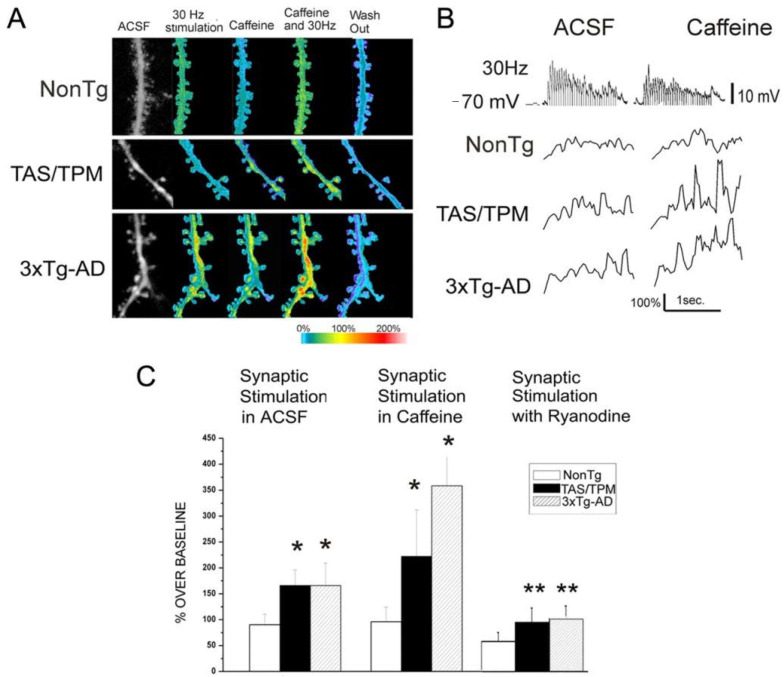
Synergistic Ca^2+^ interactions between RyR and glutamatergic synaptic transmission in AD mouse cortical neurons. (**A**) Pseudocolored images of relative Ca^2+^ changes in representative NonTg (top), TAS/TPM (a double transgenic AD mouse model) (middle), and 3xTg-AD (a triple transgenic AD mouse model) (bottom) neurons in the following conditions (from left to right): baseline 30 Hz synaptic stimulation (1.5 s), caffeine alone (10 mm), 30 Hz synaptic stimulation plus caffeine, and washout. (**B**) Representative Ca^2+^ response traces after 30 Hz synaptic stimulation (voltage trace shown in top) shown as percentage over baseline, in control aCSF (left panels) and in 10 mM caffeine (right panels) for NonTg (top), TAS/TPM (middle), and 3xTg-AD (bottom) neurons. (**C**) Bar graphs show averaged (mean ± SE) Ca^2+^ responses integrated over a 1.5 s time period of 30 Hz synaptic stimulation in control ACSF (left grouping), synaptic stimulation plus caffeine (center), and synaptic stimulation with ryanodine in the pipette (right grouping) for the NonTg, TAS/TPM, and 3xTg-AD neurons. Statistically significant differences are indicated by asterisks (one-way ANOVA, *p* < 0.05). * Significantly different from NonTg within treatment group; ** significantly different from synaptic stimulation in aCSF within transgenic strain (modified from [9]).

**Figure 2:**
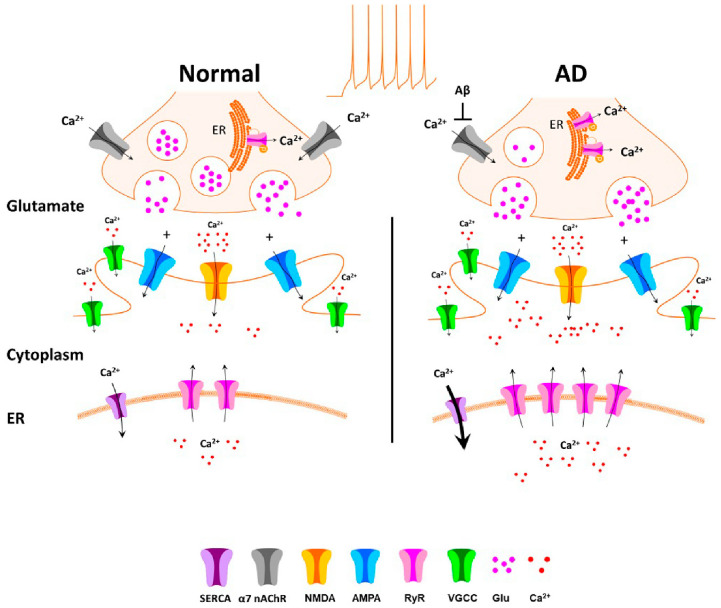
Schematic of synaptic Ca^2+^ dysregulation in AD. Under normal circumstances (left), impulse mediated increases in presynaptic Ca^2+^ result in neurotransmitter release via a ready releasable vesicular pool. In addition, activation of presynaptic α7nAChRs triggers further Ca^2+^ influx, thus facilitating impulse mediated release, and presynaptic effects may be further increased via presynaptic RyR mediated Ca^2+^-induced-Ca^2+^ release (CICR). In mouse models of AD, although intrinsic cell excitability is not increased, presynaptic RyR mediated CICR may be increased, resulting in increased release probability of glutamate and depletion of vesicle stores. Aβ binding to presynaptic α7nAChRs may result in occlusion of the binding site, with decreased function and eventual decreased presynaptic α7nAChR expression due to endocytosis. In AD, increased ER Ca^2+^ stores, along with increased RyR expression results in increased postsynaptic CICR, which may facilitate stimulus-evoked postsynaptic Ca^2+^ increases.

Mutations in the presenilin 1 (PS1) gene are linked to familial AD, an early onset form of the disease, and these mutations have specific functional implications for Ca^2+^ regulation. Although PS1 is a part of the γ-secretase complex which cleaves amyloid precursor protein (APP), it is also expressed on the ER membrane where it regulates RyR and IP_3_R channel properties [121,122,123,124], and may serve as a Ca^2+^ leak channel [125,126]. Mutations or altered expression of PS1 also affect the expression and sensitivity of neighboring RyRs [124]. RyRs play an important role in Ca^2+^ regulation, and RyR dysfunction is implicated in the Ca^2+^ dysregulation observed in AD [1]. RyR expression and RyR mediated Ca^2+^ responses are increased in the soma and dendritic spines of hippocampal and cortical pyramidal neurons of AD mice expressing PS1 mutations (Figure 1 and Figure 2) [9,30,127], effects which are normalized by acute or chronic treatment with dantrolene, a negative allosteric RyR modulator [50,114]. In particular, the RyR2 isoform, which is overexpressed in the hippocampus of AD mice [30,114], plays an important role in maintenance of synaptic function [128] and shortening of the RyR2 mean channel open time reverses the synaptic dysfunction and Ca^2+^ dyshomeostasis observed in an AD mouse model [47]. Human-induced neurons (HiN) derived from fibroblasts from AD patients expressing the PS1 mutation also display increased RyR expression and evoked RyR Ca^2+^ release [53], and RyR expression is also increased in post-mortem brains of AD patients, and patients with mild cognitive impairment [129,130]. Postsynaptic Ca^2+^ responses to high frequency stimulation (HFS) are increased in hippocampal and cortical neurons from several AD mouse models [8,9,131], effects which are mediated by RyR activation (Figure 1) [9]. Presynaptic RyR function is also increased in 3xTg AD mice and RyR activation by caffeine decreases the paired-pulse ratio of evoked CA1 field potentials to a greater extent in AD mice, as well as restoring normal frequency of spontaneously released vesicles, indicating increased facilitation of glutamate release by presynaptic RyRs [30]. Further indications of pathogenic synaptic effects resulting from altered RyR-Ca^2+^ signaling is the restoration of reduced presynaptic vesicle stores observed in AD mice back to normal levels upon treatment with Ryanodex [24].

In addition to effects on basal synaptic transmission, changes in RyR-Ca^2+^ signaling may also have implications for synaptic plasticity and LTP, which are impaired in AD mice [8,45]. High frequency stimulation (HFS) of hippocampal CA3-CA1 Schaffer collaterals, which generates LTP, initially results in a period of short-term, presynaptically mediated plasticity known as post tetanic potentiation (PTP), which results from an accumulation of presynaptic Ca^2+^ and is accompanied by an increased release probability of glutamate. This form of short term plasticity is necessary for the synaptic tagging processes involved in LTP [132], however in 3xTg AD mice, PTP is reduced, and this diminished short-term plasticity is followed by decreased LTP [8]. Chronic treatment with the RyR modulator dantrolene has been shown to restore PTP and LTP to control levels seen in non-AD mice, and this effect was accompanied by a restoration of presynaptic vesicles in the active zone, illustrating a role for aberrant presynaptic RyR-Ca^2+^ signaling in the impaired short and long-term synaptic plasticity observed in AD mice [8]. Presenilin deletion decreases Ca^2+^ effects of RyR activation, due to decreased RyR expression [121] and selective deletion of presynaptic presenilin decreases the release probability of glutamate, and LTP, an effect that is mimicked and occluded by RyR inhibition [133]. Thus, it would seem alterations in PS1 expression/function result in diminished LTP, either due to decreased or increased presynaptic RyR function, emphasizing the importance of RyR stabilization in maintenance of normal synaptic function.

Low resting cytosolic Ca^2+^ is maintained in part due to the actions of Ca^2+^-ATPases, which rapidly remove Ca^2+^ from the cell cytosol, against a concentration gradient. One of the major cellular Ca^2+^-ATPases is the SERCA pump, located on the ER membrane. SERCA function is facilitated by presenilin, and knockdown of the genes for presenilin 1 and presenilin 2 results in elevated cytosolic Ca^2+^, due to decreased SERCA mediated clearance of cytosolic Ca^2+^ [134]. Overexpression of the SERCA2b isoform typically found in neurons, and which physically interacts with the PS1 and PS2 Ca^2+^ channels on the ER membrane, results in increased Aβ [134]. Further evidence for presenilin’s role in SERCA function comes from a study showing that cells expressing a PS1 mutation show an exaggerated cytosolic Ca^2+^ response to SERCA inhibition by thapsigargin, indicating increased SERCA function [135]. SERCA inhibition also mimics the effects of selective deletion of presynaptic presenilin, on synaptic transmission and LTP [133], and inhibition of presynaptic SERCA function by cyclopiazonic acid diminished the Ca^2+^ hyperactivity observed in cortical neurons of AD mice in vivo [43]. In addition to SERCA, STIM Ca^2+^ sensors and Orai Ca^2+^ channels facilitate ER Ca^2+^ filling through the process of store-operated-Ca^2+^ entry (SOCE), a process which is deficient in PS1 mutant expressing neurons [136,137]. As ER Ca^2+^ release is elevated in AD neurons (Figure 2), in parallel with diminished SOCE activity, this opens up the possibility of an increased role for SERCA in this maladaptive pathology.

The characteristic features of AD, including maladaptive protein accumulation, increased free radicals and metabolic disruptions, are concurrent with aberrant intracellular Ca^2+^ signaling and contributes to the activation of the ER stress response in cells [138]. In an attempt to restore ER homeostasis, the ER triggers the unfolded protein response (UPR) by increasing the expression of transcription factors (ATP6c, XBPIs, and ATF4) which provides tolerance to cellular stress [139]. If the UPR is incompetent in decreasing stress, the ER triggers cell death by apoptosis [140,141] or autophagy [142]. Several animal [143,144,145] and human studies [138,146] report that AD mutations cause alterations in the UPR, thus in AD, aberrant ER-Ca^2+^ release disrupts the neuron’s compensatory mechanisms to restore cellular homeostasis and increases the vulnerability of neurons to stress and death.

## 2. Ca^2+^ Mishandling Impairs Cellular Organelle Functions in AD

In addition to synaptic signaling deficits, Ca^2+^ dyshomeostasis also has profound effects on the function of cell organelles, including the mitochondria and lysosomes, both of which play an important role in maintaining cellular and synaptic function. Like the ER, mitochondria and lysosomes act as intracellular Ca^2+^ stores, and dyshomeostasis of mitochondrial and lysosomal Ca^2+^ is emerging as a potential new source of cell dysfunction in AD, with profound implications for cellular and synaptic health.

### 2.1. Ca^2+^ Dysregulation Disrupts Mitochondrial Bioenergetics in AD

The mitochondria’s ability to buffer intracellular Ca^2+^ signaling is critical for neuronal signal transductions, ATP synthesis, and coordination with other organelles in physiological and pathological conditions. Mitochondrial dysfunction is a well-established characteristic of AD manifesting as increased free radical production and rate of oxidative damage, decreased ATP/ADP ratio and impaired bioenergetics [147,148,149,150]. Numerous differentially expressed mitochondria regulatory genes (133 in total) have been identified in the AD cohort and found that genes coding for mitochondrial oxidative phosphorylation were downregulated in both early and late AD brain specimens–specifically, NADH ubiquinone oxidoreductase subunits and complex I components which transfer electrons to the respiratory chain [151,152]. Proteomic and protein expression studies also confirmed dysregulated mitochondrial oxidative phosphorylation complexes [153] and defective enzymatic activity in the citric acid cycle and electron transport chain (ETC) [154,155].

The main function of the mitochondria is the production of ATP. Unavoidably, the by-products of electron transport in aerobic respiration are reactive oxygen species (ROS), due to electron leaks at complex I and III. Ca^2+^ overload, as is the case in AD, hinders glucose metabolism by disrupting components of the ETC such as, mitochondria complex I and II, tricarboxylic acid cycle (TCA), pyruvate dehydrogenase complex (PDHC), α-ketoglutarate dehydrogenase complex (KGDHC), malate dehydrogenase (MDH), and increasing ROS production while decreasing ATP production [156]. Redox proteomics studies identify increased oxidatively modified proteins, specifically antioxidant enzymes such as glutathione-S-transferase Mu, peroxiredoxin 6, multidrug-resistant protein 1 or 3, and GSH, in various brain regions of MCI and AD patients [157]. Additionally, enzymes involved in respiration were oxidized, specifically ATP synthase, aconitase, and creatine kinase [157]. This suggests that increased oxidative stress, as a consequence of mitochondrial Ca^2+^ overload, contributed to mitochondrial dysfunction and impaired energy metabolism in AD.

**Figure 3:**
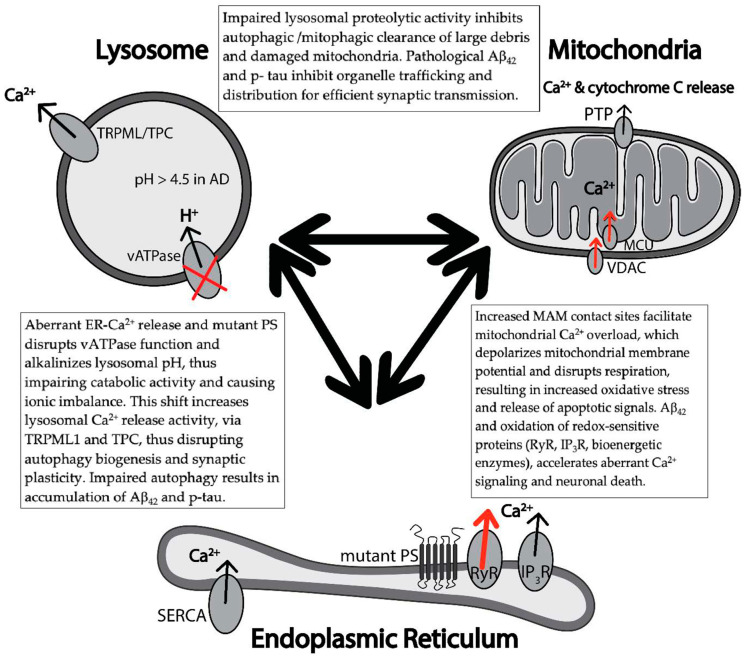
Aberrant Ca^2+^ disrupts inter-organelle functional relationships in early AD pathology. Schematic of feed-forward cascades among various neuronal Ca^2+^ handling organelles: Endoplasmic reticulutm (ER), lysosome, and mitochondria, in early AD pathology. Excess ER Ca^2+^ release through RyR and IP_3_R cause mitochondrial Ca^2+^ overload that disrupts mitochondrial bioenergetics, resulting in increased oxidative stress and apoptosis. Additionally, ER Ca^2+^ disrupts lysosome-mediated clearance of maladaptive protein deposits and damaged organelle, such as Aβ, p-tau, and mitochondria, respectively. Aberrant intracellular Ca^2+^ signaling disrupts lysosomal Ca^2+^ release via TRPML and dysregulates autophagosome biosynthesis and impairs synaptic plasticity. Presenilin (PS) mutations disrupt vATPase trafficking resulting in alkaline lysosomes, thereby disrupting lysosomal ionic balance and lysosomal Ca^2+^ store. This alkaline environment impairs proteolysis and impairs autophagic clearance.

The dynamic function of the mitochondria requires crosstalk between other major organelles, such as the ER (Figure 3). Mitochondria’s physical coupling to the ER is crucial for efficient Ca^2+^ transfer and cellular homeostasis. Mitochondrial Ca^2+^ uptake controls the rate of energy production, regulates intracellular Ca^2+^ signaling, and mediates cell death. Ca^2+^ transfer between these organelles is facilitated via the mitochondrial-associated membrane proteins (MAM). Numerous molecular proteins have been identified to support this physical interaction. Of interest, the glucose-regulated protein 75 (GRP75) is linked to IP_3_R and facilitates Ca^2+^ into the mitochondrial intermembrane space. From there, voltage-dependent anion-selective channel protein 1 (VDAC1) on the outer mitochondrial membrane, and the mitochondrial Ca^2+^ uniporter (MCU) on the inner mitochondrial membrane, transfer the Ca^2+^ to the mitochondrial matrix to stimulate the mitochondrial dehydrogenase and increase ETC activity and ATP synthesis. [158,159,160,161,162,163]. These MAMs play a crucial role in regulating mitochondrial Ca^2+^ uptake. In AD, many genes involved in mitochondrial Ca^2+^ transport are altered [164,165,166]. Of note, genes encoding mitochondrial Ca^2+^ influx, such as MCU, are downregulated whereas genes encoding mitochondrial Ca^2+^ efflux, such as NCLX, are upregulated, suggesting a compensatory mechanism to avoid excessive Ca^2+^ uptake. Additionally, studies report that, soluble Aβ aggregations increase cytosolic Ca^2+^, leading to mitochondrial Ca^2+^ overload via MCU. Excessive Ca^2+^ taken up by mitochondria leads to caspase activation and neuronal cell death [167].

In addition to Ca^2+^, VDAC1 supports transport of superoxide anions [168] and since IP_3_R and RyRs have been shown to be redox-sensitive, ROS and Ca^2+^ may play a regulatory role in ER-mitochondria communication. During AD- associated Ca^2+^ overload, mitochondrial Ca^2+^ influx elevates oxidative stress and increases ROS production. In AD, accumulated ROS has profound effects on cellular functions by oxidizing several proteins, such as the redox-sensitive RyR and IP_3_R channels. Thus, Ca^2+^ induced ROS increase and ROS-mediated Ca^2+^ increase creates a self-amplifying loop that furthers neurotoxicity and cellular dyshomeostasis, and neuronal death [169,170,171,172]. In AD, MAM proteins are shown to be associated with presenilin 1 and 2, suggesting *PSEN1/2* may alter mitochondrial Ca^2+^ transport. Additionally, increased contact sites between ER and mitochondria, via MAM proteins in AD result in elevation in ER-mitochondrial Ca^2+^ signaling and increased mitochondrial superoxide production [173,174,175,176,177,178].

Ca^2+^ is released from the mitochondria through the Na^+^/Ca^2+^ exchanger (NCLX) and the permeability transition pore [179]. The two functional states of the PTP regulates the amount of Ca^2+^ released; where the low conductance state amplifies Ca^2+^ waves and the high conductance state releases a surge of Ca^2+^ and apoptotic signals such as cytochrome C [180,181]. The biochemical signatures underlying apoptosis, rather than necrosis, indicates a choreographed and organized shutdown of the neuron. In AD, with continuous, prolonged increase in mitochondrial Ca^2+^ concentration, Ca^2+^ released from the mitochondria signals for apoptosis and increases AD pathology [172]. However the role of NCLX in AD needs further exploration as recent work suggests that impairment in glucose metabolism might reverse NCLX activity [182,183]. Additionally, impairment in NCLX accelerated memory declined and increased amyloidosis and tau pathology [184]. Mitochondrial calcium homeostasis may also rely on the activity of the plasma membrane NCLX, whose expression has been seen in differential patterns of mitochondrial expression dependent on cell type, and disruptions in expression may contribute to AD pathology [185,186,187].

Mitochondrial morphology and distribution are also crucial for neuronal homeostasis and synaptic function. Mitochondria undergo fusion and fission in the cytoplasm, which is a process to maintain a healthy pool of mitochondria with proper distribution. These mechanisms are controlled by DLP_1_ for fission and Mfn_1_, Mfn_2_, and OPA_1_ for fusion [188]. In AD, importantly, Aβ-induced and/or oxidative stress induced Ca^2+^ signaling led to increased DLP_1_ activation, resulting in excessive mitochondrial translocation and fission [189,190]. Recent studies reported that mitochondrial fragmentation, along with extensive oxidative stress and neuroinflammation, lead to neuronal loss in the cortex and hippocampus [191,192]. Excessive mitochondrial fragmentation as a result of improper Ca^2+^ handling and increased oxidative stress disrupts mitochondrial function, advancing AD pathology. Disrupted DLP_1_ and Mfn_2_ function is also responsible for reduced mitochondrial distribution. In AD, mitochondria are less abundant in neuronal processes of susceptible pyramidal neurons [193,194]. Increased tau phosphorylation negatively regulates mitochondrial movement in neurons. Tau phosphorylated at the AT8 sites inhibited mitochondrial movement in neurite processes of PC12 cells and mouse cortical neurons due to impaired microtubule spacing [195,196].

Damaged mitochondria are cleared through mitophagy, the selective degradation of mitochondria by autophagy following organelle damage or extreme cellular stress [197]. Mitophagy initiation involves the recruitment of PINK1 and PARKIN to the outer mitochondrial membrane, which tags the damaged mitochondria for degradation [198,199]. Additionally, mitophagy involves VDAC1 and MAM sensors, implicating the need for proper inter-organelle Ca^2+^ signaling and colocalization of the two organelles. Intracellular Ca^2+^ signaling relieves the inhibitory mammalian target of rapamycin (mTOR) block, thus activating mitophagy and initiating autophagosome biogenesis (ATG 32, 8, and 11) [200,201,202]. In AD patients, disruptions in mitophagy have been seen in the presence of Aβ, APP, and mutant PS1 expression. Aberrant inter-organelle Ca^2+^ signaling, as seen in AD, may disrupt degradation of damaged organelle via inactivation of lysosomal proteolysis and increase accumulation of cellular debris [198,199,203,204,205].

### 2.2. Ca^2+^ Dysregulation Impairs Lysosome-Autophagosome Mediated Protein Degradation

Lysosomal Ca^2+^ stores are responsible for regulating autophagy—a catabolic pathway utilizing the enzymatic activity of lysosomes to degrade and recycle large, bulky cellular debris, aggregated proteins, and damaged organelles. There are three types of autophagy: macroautophagy, microautophagy, and chaperone-mediated autophagy, however for simplicity, this review will focus on macroautophagy and be referred to as “autophagy”. More detailed descriptions of the aforementioned can be found in these reviews [163,206]. Autophagy is a systematic, dynamic degradation process regulated by the fusion of cargo vesicles, autophagosomes, to degradative compartments, lysosomes, with active hydrolases and proteases. While other cells rely on division to dilute cellular debris, neurons are specialized, post-mitotic cells that require efficient basal autophagy regulation to prevent accumulation of misfolded proteins and damaged organelles. Autophagy depends on the close proximity and communication between lysosomes and ER [207,208], therefore disruptions in inter-organelle Ca^2+^ signaling hinders clearance of pathological protein deposits.

Transcriptomic profiles from the collective ongoing studies known as Rush Memory and Aging Project (ROSMAP), reveal clusters of genes that are associated with pathological protein handling. Specifically, higher expression of SORL1 and ABCA7 transcripts are associated with tau tangle pathology, while elevated BIN1 transcripts are associated with beta amyloid in AD brains [209]. PLXNB1 abundance is associated with increased amyloid load and higher paired helical filaments (PHF) tau tangle density. Notably, BIN1, ABCA7, and SORL1 have functions in endocytic transport, APP metabolism and lysosome recycling, and thus are ideally positioned to serve a role in AD proteinopathy [210]. Altered expression of protein handling genes is linked to blunted endosomal trafficking, diminished degradative potential of lysosomes, and reduced autophagy-mediated clearance [210].

The key, critical feature of lysosomes is the acidic lumen (pH ~4.5) necessary for protein degradation and autophagosome digestion. The acidic pH is maintained by an active vacuolar-ATPase H^+^ pump (vATPase) driving the influx of H^+^ into the lysosome [211,212,213]. Genetic evidence linking endosomal H^+^ exchangers with AD suggest that proton leak pathways may regulate pathological Aβ generation and contribute to disease etiology [214]. In the ROSMAP AD population, there is downregulation of vATPase subunit (V1) genes, as well as the transcription factor “EB” (TFEB), a master regulator of lysosomal biogenesis that is associated with regulated autophagy [151]. Mutations in PS prevents the glycosylation, downstream maturation, and trafficking of the vATPase to the lysosome, resulting in an alkaline lysosomal lumen [215,216]. The alkaline environment inactivates protease activity, such as cathepsin B, which halts degradation of APP metabolites and dysregulates biogenesis of lysosomes and autophagosomes [217]. Additionally, cathepsins may also play a role in lysosomal trafficking along neuronal axons and dendrites, which is essential in mediating proper disposal of cellular debris. Studies showed that disrupting lysosomal proteolysis by inhibiting cathepsins or suppressing lysosomal acidification slowed axonal transport and caused selective accumulation within dystrophic neurites, a key feature of AD [218,219,220]. These are abnormally swollen regions of axons and dendrites filled mainly with autophagosomes and lysosomes, which implies improper transport of degradative organelles. Aberrant Ca^2+^ signaling, as seen in AD, can hinder lysosomal acidification and impair proteolytic enzymes in lysosomes, further AD proteinopathy and impair lysososmal trafficking, resulting in neuritic dystrophy.

Additionally, an increase in the lysosomal pH disrupts the homeostatic mechanisms to maintain the lysosomal membrane potential. The alkaline lumen depolarizes the lysosomal membrane via activation of lysosomal voltage-activated Na^+^ channels (lysoNa_V_). The Na^+^ efflux potentiates the influx of protons by the vATPase to restore lysosomal acidity. However, lysoNa_V_ are also Ca^2+^ permeable, therefore aberrant Ca^2+^ increase and changes in the Ca^2+^ concentration gradient, as seen in early pathology of AD, can hinder lysosomal acidity by reducing the driving force of H^+^ influx to restore lysosomal function [213,221,222].

This shift to a more alkaline lysosomal lumen causes hyperactivity of the lysosomal Ca^2+^ efflux channels, lysosomal transient receptor potential Ca^2+^ channel mucolipin subfamily member (TRPML1) and two-pore channel (TPC) [205,215,216,223,224,225] (although debated [226,227,228]). Lysosomal Ca^2+^ efflux through TRPML1, activates a calcineurin-dependent pathway that, via TFEB, enhances the transcription of genes involved in autophagy and lysosomal expression, such as LC3-II, ATG9B, UVRAG, WIPI, SQSTM1, MAPLC3B, GLA, GNS, HEXA, MCOLN1, TMEM55B, and ATP6V1H [229,230,231].

This lysosomal-mediated Ca^2+^ release is also responsible for fusion of autophagosomes to lysosomes. In a manner similar to neuronal vesicular fusion, lysosomal Ca^2+^ efflux channels such as, P/Q type VGCC, facilitate fusion between lysosomal tethering proteins, such as synaptogamin 7, SNAP29, and SNARE VAMP 7/8, to autophagosomal tethering proteins such as syntaxin 17 and possible SNARE proteins [232,233,234,235]. Aberrant Ca^2+^ concentration, as in AD, can influence fusion of autophagosomes to malfunctioned lysosome, resulting in accumulation of cargo vesicles with maladaptive proteins, furthering neurotoxicity.

Recent studies have shown that lysosomal functions go beyond their primary role as the degradative compartment within a neuron. Lysosomal Ca^2+^ stores are also involved in maintaining synaptic transmission. When mGluR1 is activated, NAADP-evoked lysosomal Ca^2+^ release from lysosomal Ca^2+^ channels, presumably through TPC, is amplified into Ca^2+^ waves via RyR activation [236]. This signal inactivates SK channels and prevents local hyperpolarization, which allows for greater Ca^2+^ entry through GluN receptors and facilitates the induction of LTP [237]. When VGCC is activated, NAADP-evoked lysosomal Ca^2+^ release facilitates fusion to the plasma membrane and release of cathepsin B. Cathepsin B then regulates structural plasticity and dendritic spine formation [238,239], however in pathological conditions, protease activity is inhibited and therefore synaptic dysfunctions occur. Recent work has shown that blocking endogenous cathepsin inhibitors, such as cystatin B, decreases Aβ accumulation, autophagic-lysosomal pathology, and cognitive improvement in AD mice [240].

The systemic destruction of collective organelles leads to the neuron’s demise. The close proximity of the ER to the lysosome can enhance AD pathology (Figure 3) and PS mutations may alter the RyR-lysosomes trigger zone. In healthy neurons, RyR-mediated Ca^2+^ amplification suppresses autophagic flux [207] and induces LTP [237,238,239] however, increased Ca^2+^ signaling, as seen in neurodegenerative diseases, can alter this trigger zone and therefore disrupt autophagic clearance and synaptic plasticity.
